# Effects of Awareness on Numerosity Adaptation

**DOI:** 10.1371/journal.pone.0077556

**Published:** 2013-10-16

**Authors:** Wei Liu, Zhi-Jun Zhang, Ya-Jun Zhao, Zhi-Fang Liu, Bing-Chen Li

**Affiliations:** 1 Department of Psychology and Behavioral Sciences, Zhejiang University, Hangzhou, China; 2 College of Sociology and Psychology, Southwest University for Nationalities, Chengdu, China; 3 College of Teacher Education, Ningbo University, Ningbo, China; Radboud University Nijmegen, Netherlands

## Abstract

Numerosity perception is a process involving several stages of visual processing. This study investigated whether distinct mechanisms exist in numerosity adaptation under different awareness conditions to characterize how numerosity perception occurs at each stage. The status of awareness was controlled by masking conditions, in which monoptic and dichoptic masking were proposed to influence different levels of processing. Numerosity adaptation showed significant aftereffects when the participants were aware (monoptic masking) and unaware (dichoptic masking) of adaptors. The interocular transfer for numerosity adaptation was distinct under the different awareness conditions. Adaptation was primarily binocular when participants were aware of stimuli and was purely monocular when participants were unaware of adaptors. Moreover, numerosity adaptation was significantly reduced when the adaptor dots were clustered into chunks with awareness, whereas clustering had no effect on unaware adaptation. These results show that distinct mechanisms exist in numerosity processing under different awareness conditions. It is suggested that awareness is crucial to numerosity cognition. With awareness, grouping (by clustering) influences numerosity coding through altered object representations, which involves higher-level cognitive processing.

## Introduction

Numeracy is founded upon a non-symbolic system of numerical representation, at the heart of which is the ability to perceive and discriminate numerosities [1]. Approximate estimation of number has been demonstrated in humans and other mammals [2]. Numerosity estimation is proposed to be accompanied by processes for a combination of “surrogate” features that approximate a number [3], such as total area, spatial frequency, and texture density [4–7]. Numerosity property, even when primary with a specialized attuned neural system [8–11], is not likely to be an isolated representation [12]. Instead, it should connect to other representations of magnitude. This perspective is supported by the fact that non-numerical cues can affect number processing [4,13]. In particular, changing the features of distributions of stimulus dots (e.g., when dots clustered together) profoundly decreases the perceived numerosity [4,13]. This result holds when dots are connected by lines [3,14]. 

The occupancy model provides an explanation for such decrease. Perceived numerosity is proposed to be correlated with the occupancy area defined by the sum of circles centered at each dot with certain radius. Circles overlap and estimation decreases when dots cluster [15]. However, this model cannot explain the underestimation when dots are connected because connection does not affect the occupancy area. This underestimation may reflect the perceptual organization to represent objects. Numerosity cognition may operate over a representation of objects segmented into discrete units [3,14]. According to the number–detector model, a visual image may undergo a process that isolates objects and produces a single locus of activation for each object [16]. Similarly, numerosity perception is proposed to contain the individuation of items, followed by a magnitude estimation based on distinct units [14,17]. 

Numerosity cognition may rely on the processing of both image properties and object representation [3,14]. Processes for primitive properties, which are generally analyzed in early stages of visual pathway, are necessary because numerosity must be abstracted from surrogates. Specifically, we may start with a simplified input of “blobs” of activation on a simulated retina (i.e., each stimulus is coded as an activating cluster simulating in the retina) in early stages [16]. However, the processing must eventually lead to an abstracted representation of a number, and the latter is suggested to include numerical units representation [3,14,17,18], which is proposed to involve higher-level processes [14]. In short, numerosity perception is a process that unfolds across several levels of visual processing. Our aim is to characterize how numerosity perception occurs at each level. We proposed that the potentially distinct mechanisms for each level can be analyzed by investigating the relationship between numerosity adaptation and awareness. 

Adaptation paradigm is an effective psychophysical tool for probing the neural mechanisms of numerosity processing. Adaptation to the numerical property of stimuli is usually accompanied with an aftereffect that can be revealed by changes in perceived numerosity in the following tasks [8]. This adaptation is relatively independent of texture information [2,7,8]. Liu, Zhang, & Zhao (2012) asked subjects to compare and adapt to numerosity for two types of stimuli. A total of 405 randomly distributed dots are included in one stimulus, whereas these dots clustered into 45 chunks in another. Decreases are found in both numerosity perception and adaptation when the dots clustered. Therefore, numerosity adaptation is based on the perceived numerosity rather than the real numerosity of adaptors [19]. 

The dependences of adaptation on awareness have been studied with regard to various visual properties. Awareness is relatively unimportant for adaptive coding at the lower end of ventral visual processing. However, it becomes crucial at the higher end of this pathway [20–22]. For example, the adaptive coding of density, orientation, and spatial frequency are relatively independent of awareness, whereas the coding of facial identity strongly depends on awareness [23–27]. 

As mentioned above, numerosity cognition may engage activity for several levels [3]. The coding of primitive properties primarily occurs in early stages, whereas the representation for discrete objects is proposed to occur in higher levels [28]. Importantly, low and high levels are likely to operate in parallel [16] with distinct ways of representing numerical units. Low- and high-level units (e.g., dots and objects composed of dots) can possibly mediate numerosity cognition. The representation of objects without awareness is likely to be suppressed [20]. However, the representation of discrete dots may be achieved without disruption even without awareness because it is suggested to be coded by activation on simulated retina in early stages [16]. It is possible that numerosity processing can be accomplished without awareness by selecting discrete dots as numerical units. Therefore, we hypothesize a duality in correlation with numerosity adaptation and awareness. On the one hand, numerosity adaptation may survive without awareness. On the other hand, unaware adaptation should involve distinct mechanisms if high-level processing (i.e., object representation) affects numerosity cognition with awareness and becomes suppressed without awareness.

First, numerosity adaptation with and without awareness may have distinct neural bases because distinct pathways may be involved. The difference in neural bases can be inferred by interocular transfer. Interocular transfer denotes the transfer of aftereffects between the two eyes. The quality can be investigated by comparing the adaptation of both eyes while only presenting the adaptor to one eye [29]. Monocular aftereffects essentially reflect a neural basis that involves simple cells in V1, whereas binocular aftereffects indicate a neural substrate only available for complex cells and beyond [29]. Harris et al. (2011) found that the simultaneous brightness contrast illusion, which is a monocular process based on neurons in an early stage, remains under unawareness condition. However, the Kanizsa triangle illusion, which is mediated by binocular neurons at a higher stage, vanishes [30]. The two illusions are different both in interocular transfer and awareness dependences. Similarly, the distinct neural bases for numerosity adaptation under different awareness conditions can be inferred if the interocular transfer is different in adaptation with and without awareness.

Second, the functions of object representation may differ in numerosity adaptation with and without awareness. Object representation is located in a higher stage of processing in the ventral pathway [28]. At this stage, neural mechanisms associated with awareness are highly relevant to adaptive feature coding [20,22]. Sweeny et al. (2011) suggested that the adaptive coding of open and closed curvatures straddles the divide between weakly and strongly awareness-dependent pattern coding. This finding supports the view that object representation depends on awareness because closed curvatures signal the presence of objects. A steady representation of objects will be disrupted by the absence of awareness. Therefore, mechanisms that form numerical units may be affected in the unaware adaptation. 

We proposed that mechanisms of forming objects can be inferred by “grouping effect” on numerosity adaptation [19]. The mechanisms by which clustering decreases numerosity perception and adaptation [19] have been proposed to result from the decrease in “occupancy area” [15]. However, we suspect that the underestimation reflects a mechanism of object representation. Although previous studies stated that grouping is likely to occur in multiple levels of visual organization [31], we propose that grouping affects numerosity cognition through the higher-level representation of distinct objects rather than processing of perceptual features. This hypothesis was tested in the current study. If grouping with awareness causes the decrease by changing the representation of numerical units, then the decrease should disappear in adaptation without awareness, accompanied with the suppression of object representation. By contrast, if grouping causes the decrease through primitive perceptual features (e.g., proximity or area), then the decrease should not differ whether observers adapt with awareness because processing of primitive properties is located in early stages that may survive without awareness. For example, the “occupy radius” is a product stemming from spatial filters in early analyses [15]. Based on this hypothesis, we compared the “grouping effect” on adaptation with and without awareness to infer whether the representation of objects differ under different conditions. The proposal that numerosity cognition involves the representation of discrete objects [3,14] would gain further support if our hypothesis holds.

We hypothesized that awareness is crucial in numerosity processing. Adaptation without awareness (if any exist) can reveal mechanisms different from that with awareness. First, these two adaptations may differ in interocular transfer. Second, grouping (i.e., clustering) may distinctly influence adaptation under different conditions. When observers adapt with awareness, grouping reduces adaptation [19]. As to adaptation without awareness, dot clustering may not form new objects and grouping may not decrease adaptation anymore because object representation may be constrained.

The current study combines an adaptation paradigm with a continuous flash–suppression paradigm. The status of awareness can be controlled by masking [20]. Dichoptic masking, in which masks are presented to a different eye than the adaptor, disrupts visual awareness [32–34] and maintains lateral geniculate nucleus (LGN) and V1 spiking responses to the adaptor [35,36]. By contrast, monoptic masking, in which masks and adaptors are presented to the same eye with the former superimposed on the latter, keeps the adaptor visible and disrupts LGN and V1 activities [20,37]. Generally, high-level activity is closely associated with stimulus visibility [20,38]. Therefore, dichoptic and monoptic masking influence adaptation at different levels of processing [20,38,39]. In the current study, the no-mask condition was included for comparison. The dichoptic-mask condition was conducted to investigate the adaptation without awareness. The monoptic-mask condition was included to exclusively determine the effects of awareness by controlling the potential influence of masking. Importantly, an exploration of adaptation survival under the monoptic-mask condition, where neural spiking in early stages is likely to be suppressed, is helpful to support the proposal that numerosity processing involves high-level activity [14,19]. Three experiments were conducted. In Experiment 1, we explored numerosity adaptation under three mask conditions. In Experiment 2, we compared the interocular transfer of adaptation under these conditions. In Experiment 3, we compared the “grouping effect” on adaptation under these conditions. 

## General Methods

### Ethics Statement

The data were anonymously analyzed. The subjects provided verbal and written informed consent by signing a form to receive compensation for their participation. The current study was approved by the ethics committee of the Department of Psychology and Behavioral Sciences of Zhejiang University.

### Subjects

Fifty-two right-handed volunteers (age 18–29 years) with normal or corrected-to-normal vision participated in this study. Twenty (8 male, 12 female) volunteers participated in the first experiment, sixteen (8 male, 8 female) in the second experiment, and sixteen (7 male, 9 female) in the third experiment. The participants received compensation after the experiments.

### Apparatus

The experimental stimuli were produced using Walk Script 1.0 (ZJU Walkinfo Co., Ltd., Hangzhou, China). The stimuli were presented in a dark room on a 17” flat-screen monitor with a resolution of 1024×768 pixels and a refresh rate of 85 Hz. The participants viewed the stimuli through a mirror stereoscope and responded with a keyboard. Head and chin rests were used in all experiments to ensure a consistent viewing distance of 80 cm. 

## Experiment 1: Numerosity Adaptation with and without Awareness

In Experiment 1, we adopted three mask conditions to examine the numerosity adaptation with and without awareness: no-mask, monoptic-mask, and dichoptic-mask conditions. Participants under the no-mask condition observed adaptors without any masks. Adaptors and masks were presented to the same eye (non-dominant eye) under the monoptic-mask condition, wherein the latter was superimposed on the former. Masks were presented to a different eye (dominant eye) than the adaptors under the dichoptic-mask condition. The participants were generally aware of the adaptors under the no-mask and monoptic-mask conditions and unaware under the dichoptic-mask condition. The status of awareness for adaptors under the monoptic- and dichoptic-mask conditions was estimated by subsequent tests. We adopted a within-participant design in all three experiments. 

### Materials and Methods

#### Stimuli

**Figure 1:**
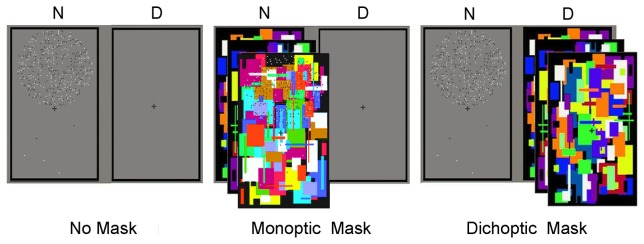
Adaptor and masks used in Experiment 1. The adaptors were shown to the non-dominant eye. A gray background with a centered fixation cross was presented to the dominant eye during the adaptation stage under the no-mask condition. A continuous flash suppression (CFS) with a centered fixation cross was shown to the non-dominant eye while a similar gray frame was presented to the dominant eye during adaptation under the monoptic-mask condition. A CFS with a fixation was shown to the dominant eye during adaptation under the dichoptic-mask condition. “N” represents the non-dominant eye, and “D” represents the dominant eye in the figure.

All stimuli were displayed using two binocular frames with black checkerboard borders consisting of 9.43°×15.52° gray rectangles with thicknesses of 0.18° (Figure 1). These frames used a gray background and remained on the screen throughout the entire trial sequence. The adaptors or tests were presented in one of the frames. The mask stimuli were assigned to the same frame under the monoptic-mask condition and assigned to the other frame under the dichoptic-mask condition. The square dots (0.13°×0.13°) were generated and randomly distributed within two fixed circles with diameters of 7.15° and centers at 3.94° above or below the center of the frame. Half of the dots were white, and the others dots were black. For the adaptor, 405 dots were arranged in one circle, and 5 dots were arranged in the other circle. The mask stimuli were composed of colored, irregularly distributed rectangles that covered each other. 

### Design and Procedure

Participants initiated the first trial by pressing the space bar. Adaptors were presented to the non-dominant eye in the adaptation stage. The continuously flashing mask stimulus was superimposed on adaptors and presented to the same eye under the monoptic-mask condition. Masks were presented to the dominant eye to prevent participants from consciously perceiving the adaptor under the dichoptic-mask condition. A gray background with a centered fixation cross was presented to the dominant eye under the no-mask condition. The testing stage used a point of subjective equality (PSE) to quantify the perceived numerosity; the numerosity adaptation aftereffects were measured by subtracting the PSEs before from the PSEs after adaptation [29]. We used a constant stimulus method to quantify PSE. Two sets of dots were successively shown in the two fixed circles, and each trial provided all participants with a forced choice question: “Which circle contained more dots?” The constant stimulus (i.e., the probe) contained 30 dots. The PSEs of adaptation under the different mask conditions were estimated with different responding serials in preparation experiments to determine the range of comparison stimuli (i.e., test). A rough range of 40 to 50 can be determined for PSEs with adaptation under the monoptic- and dichoptic-mask conditions. Finally, the tests were fitted as 20, 27, 33, 40, 49, 60, 74, and 90 dots. The quantities of the compared stimuli were chosen using an equidistance logarithm scale [40] with a center of 40 to 50 rather than the probe number (30) because we considered the effects of adaptation. We propose that the serial comparison can ensure an approximately equal number of top and bottom presses under the monoptic- and dichoptic-mask conditions (with adaptation), which were the major interests in the current study. The serial might slightly shift PSEs without adaptation (i.e., PSEs for baselines) under all conditions. However, we argue that the influence can be counterbalanced because the influence was identical for every baseline. Six equivalent stimuli were provided for the probe (each containing 30 randomly spreading dots), and four equivalent stimuli were provided for each test. A total of 24 paired stimuli were generated when we matched the probe to the test; half were randomly chosen for the experiment, and 96 comparison judgments were made.

**Figure 2:**
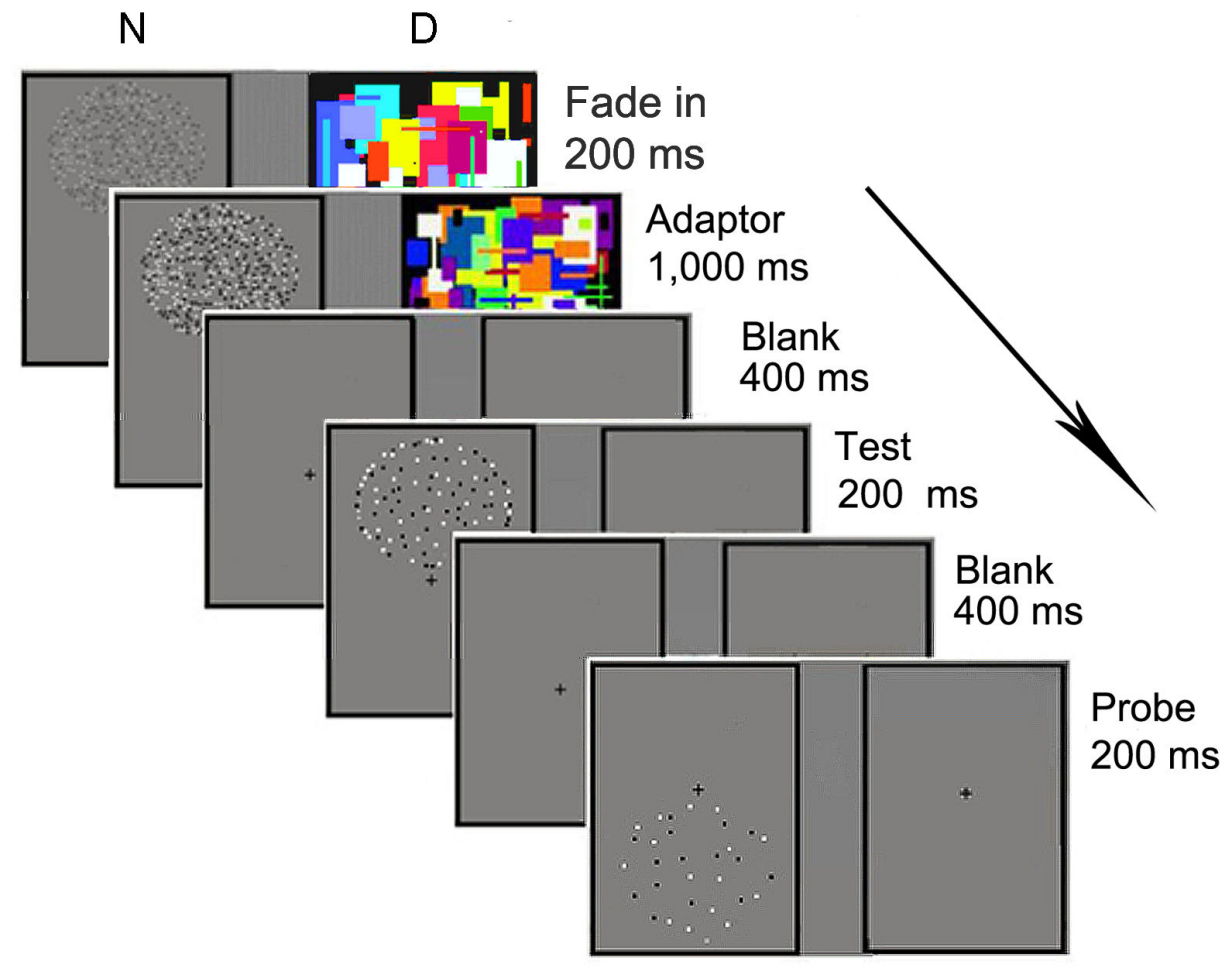
Schematic of the paradigm in Experiment 1. We used adaptation in the dichoptic-mask condition (right-eye-dominance) as an example. Each trial began with a top-up adaptation stage (Figure 1) that lasted for 1,000 ms when adaptors had faded in (during 200 ms) to one frame, while masks flashed on the other frame. The test stage began with a test stimulus displayed in the same position as the 405-dot adaptors for 200 ms, followed by a probe stimulus displayed directly below for 200 ms. All stimuli were separated by a blank screen for 400 ms. The participants viewed the stimulus with both eyes. They kept their eyes on the fixation cross during the trial, reported whether the probe appeared more or less numerous than the test, and guessed when unsure.

The procedure (with adaptation, right-eye-dominance) of the dichoptic-mask condition is described in Figure 2. The adaptors in the adaptation stage faded in to the left frame across four steps, each of which lasted for 50 ms. Then, the dots remained there for 1,000 ms. Twelve masks flashed in the right frame for 100 ms per mask during the entire procedure. In the testing stage, a fixation cross was shown in the left frame for 400 ms, followed by a test presented for 200 ms in the above circle . Then, the probe was shown in the below circle for 200 ms. A blank frame with a fixation cross isolated the test and probe for 400 ms. The next trial began either after a participant’s response or 1,000 ms without a response. A stable gray background with a fixation cross was presented in the right frame during the testing stage. All stimulus positions were horizontally reversed for participants with left eye dominance.

The Miles test (http://en.wikipedia.org/wiki/Ocular_dominance) for eye dominance was conducted prior to the experiments [41]. Then, participants completed three pretests prior to adaptation tasks to create adaptation aftereffect baselines that were measured as controls under the corresponding mask conditions. The baseline under the no-mask condition was obtained from a task identical to the procedure of the testing stage mentioned above. The baselines under the monoptic- and dichoptic-mask conditions were obtained from tasks that were quite similar to the previously described procedure, except that no adaptors were presented when masks flashed in the left (monoptic mask) or right (dichoptic mask) frames during the “adapting” stage. A brief practice stage with feedback was added to improve participants’ familiarity prior to all formal experiments. The stimulus positions (top or bottom) were reversed for half of the participants in Experiment 1 to counterbalance the probable influence of retinal position asymmetry. 

The status of participant awareness to the adaptor was examined after the adaptation tasks under the monoptic- and dichoptic-mask conditions. The participants answered a forced-choice question (“top or bottom?”) to identify the adaptor position (405 dots) for 80 trials. These stimuli were presented similarly to former tasks. The data for participants who perceived the adaptor under the dichoptic-mask condition were not included in formal analysis. 

### Results and Discussion

The average correct ratio of the position-deciding task under the dichoptic-mask condition was 51.6%, with a maximum of 53.9% and a minimum of 43.2%. The average correct ratio was 98.6% under the monoptic-mask condition. All participants had orally reported no detection of any dots (adaptors) under the dichoptic-mask condition and clearly perceived the adaptors under the no-mask and monoptic-mask conditions.

**Figure 3:**
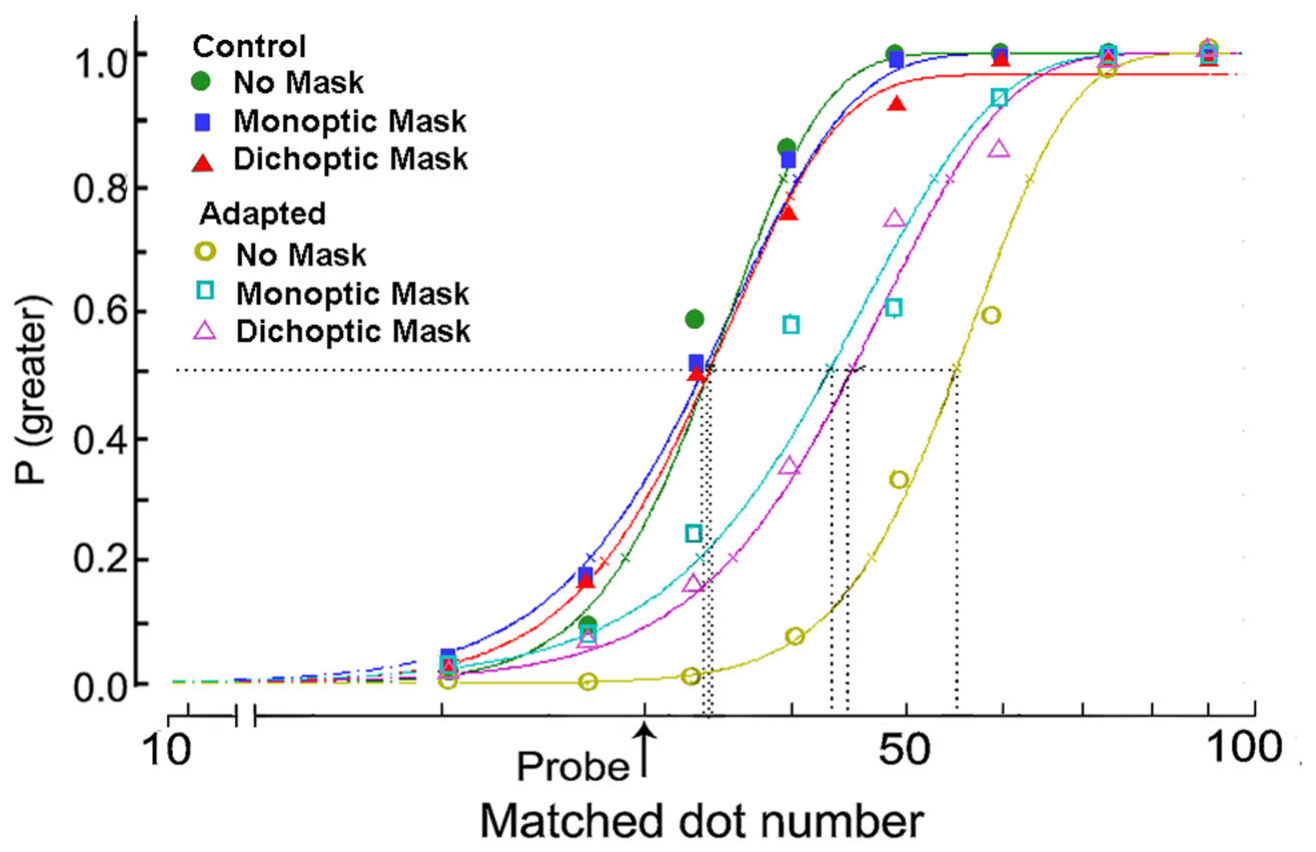
Samples of psychometric functions under different conditions in Experiment 1. The proportion of trials is plotted such that the probe seemed more numerous as a function of the number of test dots. The fitted results from one participant were shown as examples. The vertical dashed lines indicate the PSEs (i.e., the point of subjective equality) of the match, which were clearly higher than the probe number (indicated by the arrow) when adapted under the no-mask (open circles), monoptic-mask (open rectangles), and dichoptic-mask (open triangles) conditions. The PSEs with adaptation were significantly greater than their respective baselines (filled circles=no-mask condition; filled rectangles=monoptic-mask condition; filled triangles=dichoptic-mask condition). Adaptation decreased under the monoptic- and dichoptic-mask conditions compared with adaptation under the no-mask condition.

Cumulative normal functions were fitted to the psychometric functions of each participant using the psignifit toolbox version 2.5.41 for Matlab (http://www.bootstrap-software.com/psignifit/) to implement the maximum-likelihood method described by Wichmann and Hill (2001) and thus quantitatively measure the magnitude of the adaptation aftereffect [42]. The 50% points of the fitted functions were obtained (Figure 3). The values of the test stimuli (X axis) corresponding to the 50% points were calculated from the fitted curves. These values were PSEs representing the number of test dots, which appeared to equal the number of probe dots for participants. Therefore, the change in numerosity perception in the tests can be demonstrated by the difference in the average values for PSEs under different conditions (Table 1). The magnitude of the numerosity adaptation aftereffect can be indicated by the PSEs under adaptation conditions minus the PSEs under their control conditions (baselines) [29].

**Table 1:** Mean and SD for PSEs for different groups in Experiment 1.

	**No mask**		**Monoptic mask**		**Dichoptic mask**	
**Treatment**	**Control**	**Adapted**	**Control**	**Adapted**	**Control**	**Adapted**
**PSE**	33.87	54.37	34.18	41.39	34.58	43.21
**SD**	2.17	7.87	4.63	4.70	4.04	6.90

Note. PSE: point of subjective equality of participants when they decided the numerosity of the probe. SD: standard deviation of PSE.

**Figure 4:**
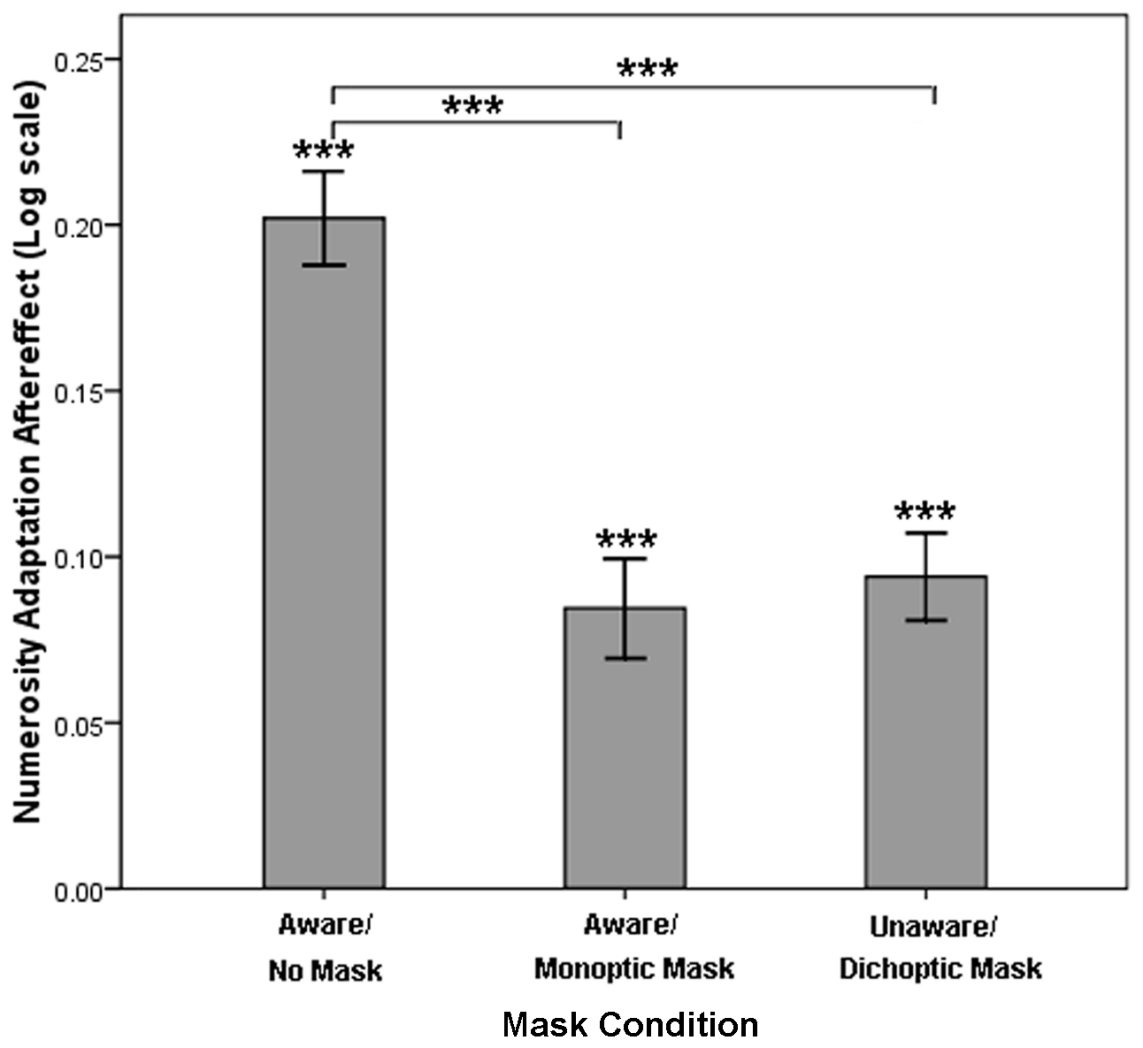
Results from Experiment 1. A one-way analysis of variance (ANOVA) with mask condition (no mask, monoptic mask or dichoptic mask) as the independent variable and adaptation aftereffect as the dependent variable was conducted in Experiment 1. The adaptation aftereffects are shown in log scale. The asterisks indicate statistical significance (***p<.001), and the error bars denote 1 standard error of the mean (SEM).

Significant differences in the PSEs were not found in the baseline scores (*p*>.05). This finding suggests that the masks did not affect quantity judgments. Numerosity adaptation showed a significant aftereffect under the no-mask condition because the PSE with adaptation differed from its baseline, *t*(19) = 12.15, *p*<.001. The adaptation was also significant under the monoptic-mask condition compared with its baseline, *t*(19) = 5.81, *p*<.001, indicating that numerosity adaptation survived when neural activities in the early stage were likely to be disrupted [20]. Notably, the adaptation was still significant under the dichoptic-mask condition compared with its baseline, *t*(19) = 6.97, *p*<.001, demonstrating that participants adapted to the adaptor without conscious adaptor perception. The results of a one-way analysis of variance (ANOVA) with mask condition (no mask, monoptic mask, or dichoptic mask) as the independent variable and adaptation aftereffect as the dependent variable revealed that the mask condition showed a significant effect (Figure 4), *F*(2, 18) = 22.63, *p*<.001, *η*_p_^2^=.72. The adaptation aftereffects in the no-mask condition was significantly different from that in the monoptic-mask condition (*p*<.001) and that in the dichoptic-mask condition (*p*<.001), whereas no significant difference was found between the monoptic- and dichoptic-mask conditions. 

Experiment 1 showed that adaptation decreased but still survived under both monoptic- and dichoptic-mask conditions. Suppression was likely to have been occurring in early-stage processing under the monoptic-mask condition, whereas high-level activities were profoundly disrupted under the dichoptic-mask condition, accompanying with the elimination of awareness for adaptors [20,38]. In short, numerosity adaptation can survive when either low- or high-level processing was suppressed. These results indicate a duality in the numerosity processing mechanism: both low-level and high-level pathways can be engaged in parallel to accomplish numerosity cognition. In addition, distinct pathways were involved under different mask conditions. In other words, we proposed that numerosity adaptation engaged high-level processing under the monoptic-mask condition, whereas adaptation was primarily based on low-level activity under the dichoptic-mask condition. Naturally, we cannot exclude the possibility that low-level activity accounted for the reduction in adaptation under monoptic- and dichoptic-mask conditions because the masking and the lack of awareness might have similar effects on low-level activity [20]. Investigating the characteristics of adaptation under different mask conditions is warranted to decide whether different mechanisms are engaged in adaptation of different mask conditions. 

## Experiment 2: Interocular Transfer of Numerosity Adaptation

Significant adaptation aftereffects were found in both the awareness (no mask and monoptic mask) and unawareness (dichoptic mask) conditions in Experiment 1. Experiment 2 examined the interocular transfer of numerosity adaptation in three mask conditions to infer the neural bases of numerosity adaptation across different awareness conditions.

### Materials and Methods

The materials used in Experiment 2 were similar to Experiment 1. The adaptation aftereffects in the same and different eyes were measured in three mask conditions, respectively, while the adaptors were solely assigned to one eye. Specifically, the adaptors were only presented to the non-dominant eye. The probe and tests were both presented in the dominant and non-dominant eyes, and they were just presented to a single eye in each treatment. Each participant completed all treatments and pretests (controls). The pretests in Experiment 2 were referred to the non-dominant and dominant eyes. The adaptation treatments under the monoptic- and dichoptic-mask conditions were conducted in a random order. Adaptations with no mask were conducted later because their aftereffects could be much larger. We used this sequence to avoid additional treatment aftereffects. The participants rested between treatments in an additional attempt to eliminate the influence of treatment aftereffects. Similar to Experiment 1, the perceptual status of the adaptor was examined when participants finished the monoptic- and dichoptic-mask (adaptation) tasks. 

### Results and Discussion

The average ratio of correct positions in the deciding task for the dichoptic-mask condition was 49.6%, with a maximum of 54.7% and a minimum of 43.8%. The average correct ratio was 98.2% under the monoptic-mask condition. Each participant reported an unawareness of adaptors under the dichoptic-mask condition and a clear awareness under the no-mask and monoptic-mask conditions.

**Figure 5:**
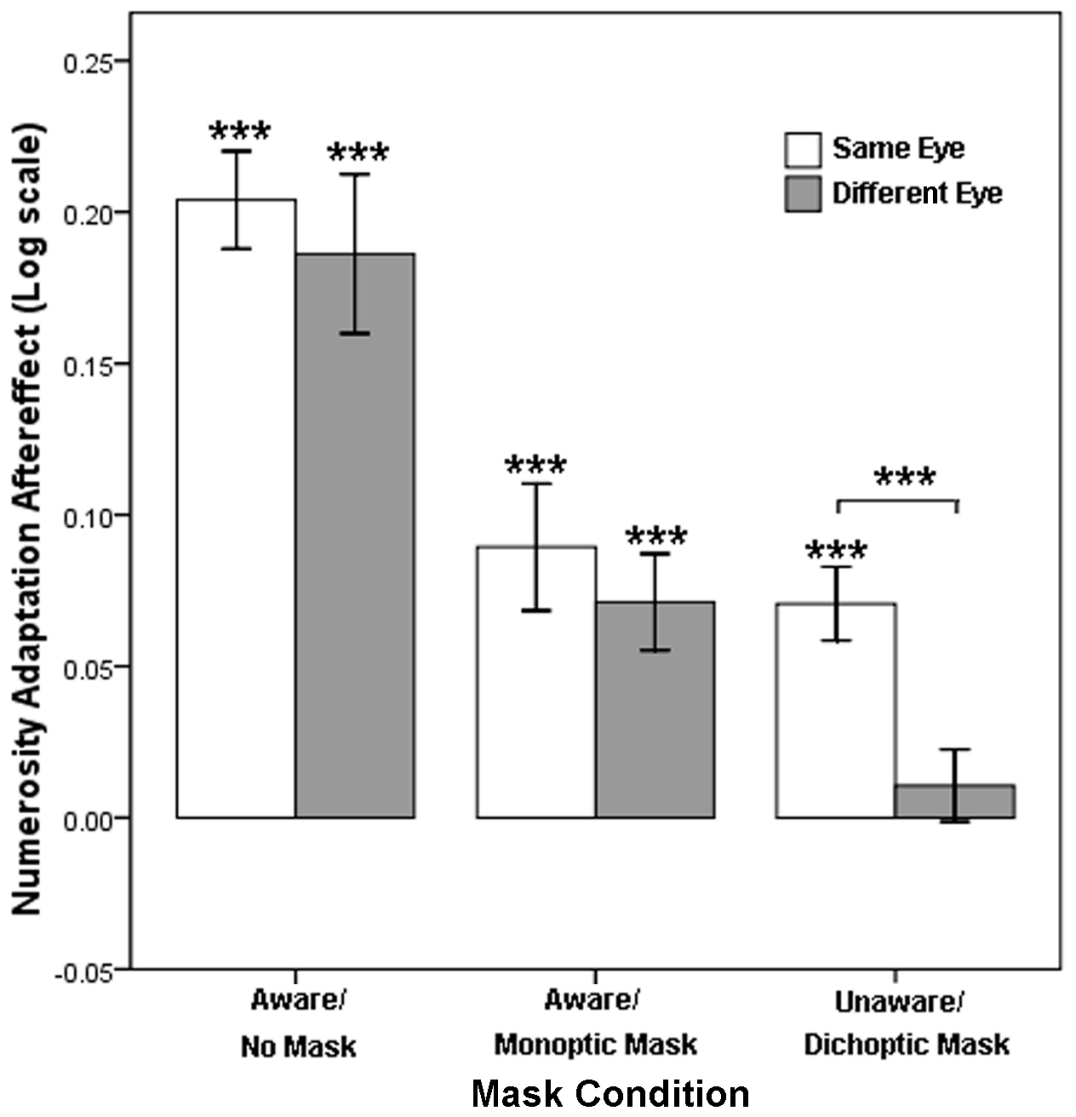
Results from Experiment 2. A 3×2 repeated measured ANOVA with mask condition (no mask, monoptic mask, or dichoptic mask) and tested eye (same or different) as the independent variables and adaptation aftereffect as the dependent variable was conducted. The adaptation aftereffects are shown in log scale. Asterisks indicate statistical significance (***p<.001). Error bars denote 1 SEM.

Table 2 shows the calculated results of average PSEs. Under the no-mask condition, significant adaptation aftereffects were found in the same eye (i.e., tested eye to which the adaptors were presented), *t*(15)=9.66, *p*<.001, and the different eye (i.e., tested eye to which the adaptors were not presented), *t*(15)=6.64, *p*<.001. Under the monoptic-mask condition, significant adaptation aftereffects were found in the same eye, *t*(15)=4.48, *p*<.001, and the different eye, *t*(15)=4.51, *p*<.001. Under the dichoptic-mask condition, a significant effect was found in the same eye, *t*(15)=5.93, *p*<.001, but not in the different eye (*p*>.05). A 3×2 repeated measured ANOVA was conducted with mask condition (no mask, monoptic mask, or dichoptic mask) and tested eye (same or different) as the independent variables and adaptation aftereffect as the dependent variable (Figure 5). This model demonstrated significant effects of mask condition, *F*(2, 14)=29.76, *p*<.001, *η*_p_^2^=.81, and tested eye, *F*(1, 15)=8.06, *p*<.05, *η*_p_^2^=.35, as well as a significant interaction between these factors, *F*(2, 14)=4.45, *p*<.05, *η*_p_^2^=.39. Significant differences were not found between the same and different eyes when participants adapted with no masks (*p*>.05) or with monoptic masks (*p*>.05), whereas a significant difference was observed when participants adapted with dichoptic masks, *F*(1, 15)=37.56, *p*<.001, *η*_p_^2^=.72. 

**Table 2:** Mean and SD for PSEs for different conditions in Experiment 2.

	**No mask**				**Monoptic mask**				**Dichoptic mask**			
**Tested eye**	**Same Eye**		**Different Eye**		**Same Eye**		**Different Eye**		**Same Eye**		**Different Eye**	
**Treatment**	**C**	**A**	**C**	**A**	**C**	**A**	**C**	**A**	**C**	**A**	**C**	**A**
**PSE**	33.50	54.31	34.30	53.19	34.71	42.53	35.44	41.74	35.63	42.07	35.60	36.50
**SD**	1.82	9.79	3.78	9.84	4.54	4.71	3.21	3.75	3.10	5.00	3.15	3.48

Note. PSE represents the point of subjective equality when participants decided the numerosity of the probe. SD represents the standard deviation of PSE. “C” refers to the control conditions, and “A” refers to the adapted conditions.

When adaptors were solely presented to one eye, the mask conditions influenced the interocular transfer of numerosity adaptation. With awareness (no-mask and monoptic-mask conditions), adaptation aftereffects did not differ between the same and different eyes, indicating that the effect was primarily binocular. Without awareness (dichoptic-mask conditions), the aftereffects were distinct in two eyes, and no significant aftereffects were found in the different eye, indicating that the effect was purely monocular. These results indicate that awareness should account for the interocular transfer of numerosity adaptation. The difference in interocular transfer indicated the distinct neural bases of numerosity processing with and without awareness.

Interocular transfer was only investigated from the non-dominant eye to the dominant eye in Experiment 2. However, the results did not obviously differ when we investigated interocular transfer from the same eye to a different eye (i.e., a mixture of non-dominant and dominant eyes, see 29), which are not formally reported here. 

## Experiment 3: Effects of Grouping on Numerosity Adaptation

Experiment 3 went a step further to determine the effects of grouping (i.e., dots clustered into chunks) on numerosity adaptation, and effects were investigated under three mask conditions. We sought to reveal the crucial function of object representation in numerosity cognition.

### Materials and Methods

**Figure 6:**
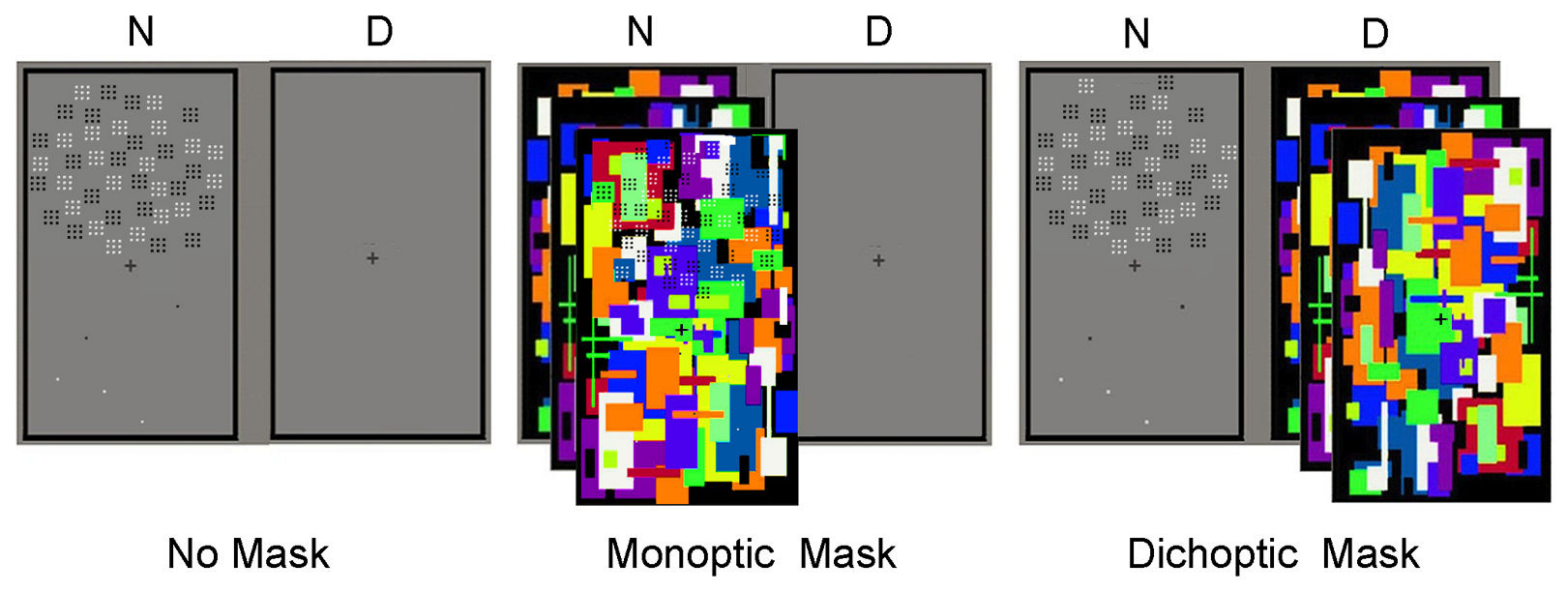
New adaptor and masks used in Experiment 3. Participants can organize 405 dots into 45 chunks by perceptual grouping, and the chunks were randomly spread within the same circle used in the previous experiments. The new adaptors (i.e., chunks) under the three mask conditions are shown in the figure. The 405-dot adaptor (Figure 1) was additionally used for comparison. The contrast for the 45-chunk and the 405-dot adaptors was 50% of the contrast for the adaptors used in the previous experiments. “N” represents the non-dominant eye, and “D” represents the dominant eye in the figure.

Similar to previous experiments, 405-dot adaptors were used in Experiment 3. In addition, 405 dots were chunked to form a new adaptor that contained 45 chunks, and each chunk was composed of nine dots (all black or all white, Figure 6). The contrasts of the 405-dot adaptors and 45-chunk adaptors were lowered by 50% compared with Experiments 1 and 2 because of the prominent decrease in perceptual thresholds when the adaptor dots were organized into chunks. We adopted orders similar to Experiment 2 for different treatments, and the participants were asked to rest between treatments. Three control pretests were conducted before the adaptation tasks, and the perceptual status of the adapting stimulus was tested after the participants finished the monoptic- and dichoptic-mask (adaptation) tasks. 

### Results and Discussion

The average correct ratios of the position-deciding tasks under the dichoptic-mask conditions were 49.6% (dots or chunks, maximum=51.4%, minimum=41.9%). The average correct ratio was 97.9% under the monoptic-mask condition (dots or chunks). Each participant reported an unawareness of adaptors under the dichoptic-mask condition and a clear awareness under the no-mask and monoptic-mask conditions. 

Table 3 shows the calculated results of the average PSEs. Significant adaptation aftereffects were found when the participants adapted to both adaptors under all three mask conditions; in the no-mask condition, to 405 dots, *t*(15)=7.27, *p*<.001 and to 45 chunks, *t*(15)=5.16, *p*<.001; in the monoptic-mask condition, to 405 dots, *t*(15)=4.95, *p*<.001 and to 45 chunks, *t*(15)=4.18, *p*<.01; in the dichoptic-mask condition, to 405 dots, *t*(15)=5.26, *p*<.001 and to 45 chunks, *t*(15)=4.16, *p*<.01. No significant difference in adaptation aftereffects was found between Experiments 1 and 3 under the no-mask condition, suggesting that the reduction in contrast did not primarily affect numerosity adaptation, which was consistent with Burr and Ross (2008).

**Table 3:** Mean and SD for PSEs for different conditions in Experiment 3.

	**No mask**			**Monoptic mask**			**Dichoptic mask**		
**Treatment**	**Control**	**Dots**	**Chunks**	**Control**	**Dots**	**Chunks**	**Control**	**Dots**	**Chunks**
**PSE**	33.97	52.54	42.81	34.55	43.09	39.12	34.05	40.41	41.00
**SD**	3.05	9.60	5.61	2.60	7.49	4.41	2.44	4.20	5.73

Note. PSE is the point of subjective equality of participants when they decided the numerosity of the probe. SD is the standard deviation of PSE. “Dots” refers to the treatment of adapting to 405 dots, and “Chunks” refers to the treatment of adapting to 45 chunks, respectively.

**Figure 7:**
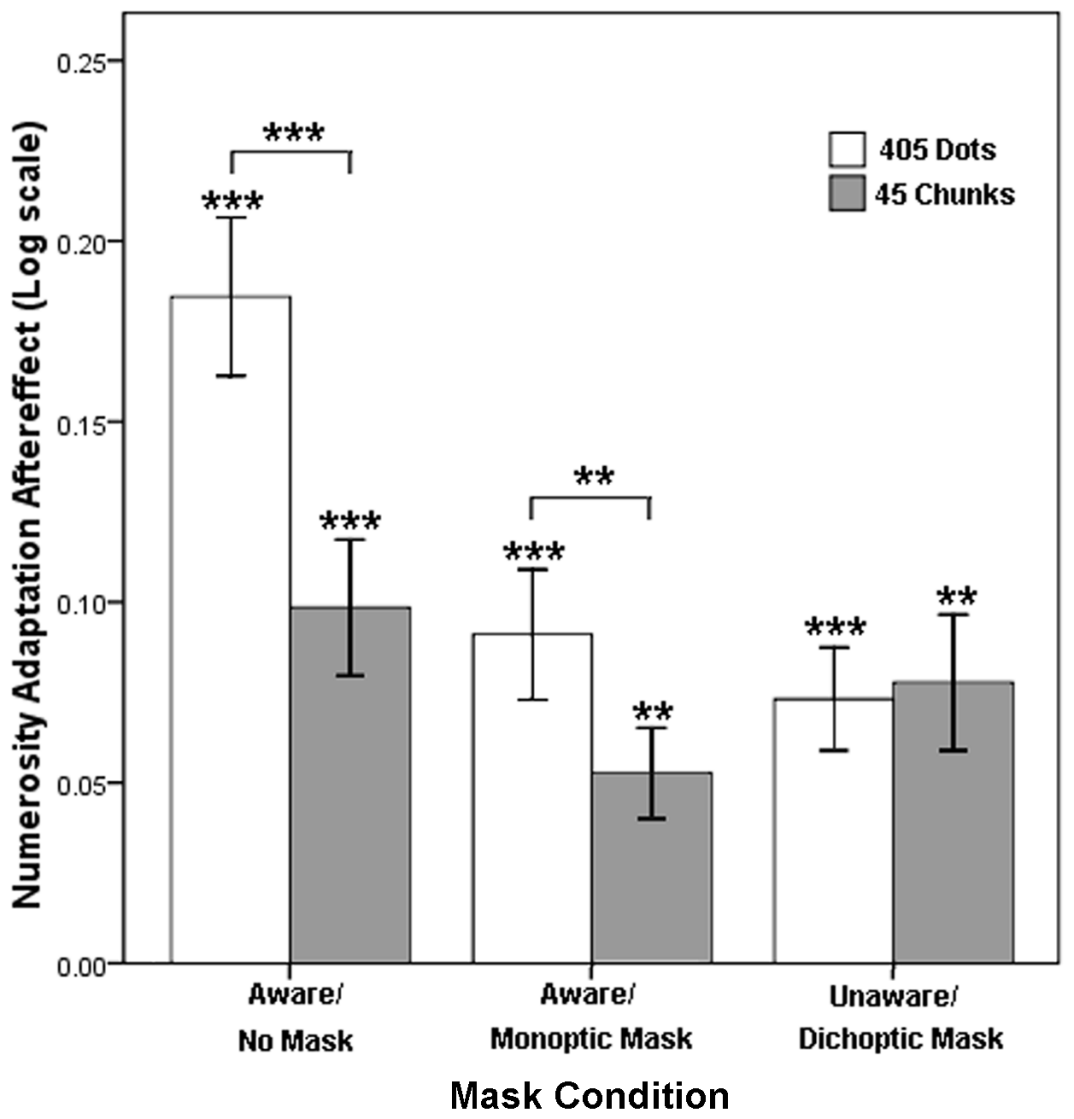
Results from Experiment 3. A 3×2 repeated measured ANOVA with mask condition (no mask, monoptic mask, or dichoptic mask) and adaptor (dots or chunks) as the independent variables and adaptation aftereffect as the dependent variable was conducted. The adaptation aftereffects are shown in log scale. Asterisks indicate statistical significance (**p<.01, ***p<.001). Error bars denote 1 SEM.

A 3×2 repeated measured ANOVA was conducted with mask condition (no mask, monoptic mask, or dichoptic mask) and adaptor (chunks or dots) as the independent variables and adaptation aftereffect as the dependent variable (Figure 7). This model yielded significant effects under the mask condition, *F*(2, 14)=7.86, *p*<.01, *η*_p_^2^=.53 and the adaptor, *F*(1, 15)=18.51, *p*<.001, *η*_p_^2^ =.55, and a significant interaction was found between these factors, *F*(2, 14)=15.51, *p*<.001, *η*_p_^2^ =.69. Significant differences were found between adaptors when participants adapted under the no-mask, *F*(1, 15)=27.95, *p*<.001, *η*_p_^2^=.65, and the monoptic-mask, *F*(1, 15)=8.72, *p*<.01, *η*_p_^2^=.37, conditions. However, no significant difference was found between adaptors when participants adapted under the dichoptic-mask condition (*p*>.05). 

The results of Experiment 3 demonstrated that grouping had distinct effects on numerosity adaptation when participants adapted under different mask conditions. Grouping strongly decreased the numerosity adaptation aftereffects when participants were aware of adaptors. However, the adaptation aftereffects did not differ when the participants were unaware of adaptors, regardless of whether the stimulus dots were grouped. “Grouping effect” disappeared with the elimination of awareness. A plausible explanation is that grouping without awareness did not form new numerical units rather than discrete dots, which should underlie the numerosity perception and adaptation. 

## General Discussion

### Distinct mechanisms exist in numerosity adaptation across different awareness conditions

 In our study, the adaptation of non-symbolic numerosity properties was investigated with and without participants’ awareness. In Experiment 1, adaptation under the no-mask condition showed a significant aftereffect with a brief adaptation stage. Aftereffects decreased but still survived under the monoptic- and dichoptic-mask conditions. Low-level neural activity is likely to be inhibited under the monoptic-mask condition [20,38,39], whereas the disruption of high-level activity can be inferred based on the fact that adaptor awareness was eliminated under the dichoptic-mask condition [20,38]. Numerosity adaptation can survive when either low- or high-level processing is likely to be suppressed [20]. In the present study, we suggested that both low-level and high-level pathways might be engaged in parallel to accomplish numerosity cognition and that distinct pathways were engaged under different mask conditions. However, we could not exclude the possibility that both the masking and absence of awareness have effects on low-level activity in Experiment 1. The distinct characteristics of adaptation under different mask conditions, which were revealed in the following experiments, support the distinction of adaptation surviving under monoptic- and dichoptic-mask conditions. 

First, we examined the adaptation aftereffects in the same eye (i.e., the eye exposed to the adaptor during the adapting stage) and the different eye (i.e., the eye that was not exposed to the adaptor) in Experiment 2. The results suggested that awareness had distinct influences on the interocular transfer of adaptation under different awareness conditions. With awareness (no-mask and monoptic-mask conditions), adaptation was primarily binocular. Without awareness, adaptation remained solely in the same eye (i.e., monocular adaptation) under the dichoptic-mask condition. Difference in interocular transfer indicates distinct neural bases in numerosity cognition under the different conditions. Monocular aftereffects reflect a neural basis that cannot be generated interocularly such as simple cells in V1, whereas binocular aftereffects indicate a neural substrate only available for complex cells and beyond [29]. The results of Experiment 2 showed that numerosity adaptation without awareness (dichoptic-mask condition) is a monocular process based on neurons in an early stage of processing and that adaptation with awareness (no-mask and monoptic-mask conditions) should engage activity of binocular neurons [29]. 

 Second, we investigated the effects of grouping on numerosity adaptation with and without awareness in Experiment 3. A significant interaction between mask condition and adaptor was found. Although the number of stimulus dots was held constant, the adaptation decreased sharply when the dots were clustered into chunks with awareness (no-mask and monoptic-mask conditions), whereas the adaptation was unaffected when the same clustering was conducted without awareness (dichoptic-mask condition). The mechanisms underlying the “grouping effect” under the different mask conditions would be discussed in detail in the third section. Nevertheless, an expressive distinction in numerosity adaptation with and without awareness can be inferred. 

 In conclusion, numerosity adaptation with awareness (no-mask and monoptic-mask conditions) was primarily binocular, suggesting that numerosity processing is based on binocular neural activity [29]. The adaptation was significantly decreased by clustering of adapting dots, underlining the function of grouping in numerosity cognition with awareness. By contrast, the adaptation without awareness (dichoptic-mask condition) appeared to be purely monocular, indicating a neural basis composed of a monocular population of neurons involving simple cells in V1 [29]. The adaptation was not sensitive to grouping without awareness, which suggested that grouping was not involved in numerosity processing when awareness was absent. The different adaptation characteristics that were revealed across awareness conditions in Experiments 2 and 3 demonstrated their respective neural bases and processing mechanisms. In other words, different mechanisms were engaged in affecting adaptation between the monoptic mask and the absence of awareness. The proposal that both a low- and a high-level processing can be engaged in parallel to accomplish numerosity cognition [16] was supported. This point of view would be further discussed in the following sections. 

### Awareness may selectively affect the high-level processing of numerosity

 We argued that distinct mechanisms underlay numerosity cognition with and without awareness. We propose an explanation for the distinct features; specifically, a series of processing may be involved in numerosity cognition, and some series could be selectively affected by awareness.

The coding and adaptation of visual properties across different levels have a range of different mechanisms in the ventral pathway, ranging from mechanisms that depend on low-level neural activities to mechanisms that depend on high-level neural processes. The latter is associated with awareness [20,24–27]. Numerosity representation is not necessarily a single event; rather, a series of processing steps or pathways, some of which can be selectively affected by awareness, may be involved. Thus, the processing of numerosity differs across different awareness conditions. This view is well supported by the number–detector model proposed to account for the cortical extraction of quantitative information from sensory inputs [16]: Two steps implement numerosity processes in parallel; the stimuli are coded as local Gaussian distributions, accompanied with a normalization of these items into size-independent summation clusters. The summation clusters finally project into numerosity clusters. In this model, both low- (e.g., retinotopic mapping) and high-level processes (e.g., object representation and the position coding) work to implement quantity representations [43,44]. This model shed light on the mechanism by which awareness influences numerosity processing. We speculate that awareness primarily affects the second step because the first stage occurs in an early sensory coding stage. Furthermore, connection, clustering, and other analogous factors shown to profoundly influence numerosity processing [3,14,44] may also affect quantity perception in the second stage. 

In fact, we go a step further to hypothesize that awareness influences numerosity processing by affecting object representation, which is processed at a higher level [20]. In particular, changing the representation of the numerical units decreased numerosity perception and adaptation when dots were clustered into chunks with awareness. However, the mechanism of forming new objects (i.e., numerical units) was proposed to be disrupted in the absence of awareness [20,30]. In other words, grouping (clustering of dots, with awareness) should affect numerosity processing by affecting object representation. We would discuss this process in detail in the following section.

### Grouping affects numerosity processing by affecting object representation

Liu et al. (2012) found that grouping (with awareness) significantly affects both numerosity perception and adaptation. The participants significantly underestimated the dots’ number and tended to directly respond to the number of chunks when the stimulus dots were clustered into chunks. In addition, a new adaptation based on the number of chunks formed automatically, which can be demonstrated by comparing the adaptation aftereffect of the chunks (e.g., 45 chunks) with that of the dots with the same quantities (e.g., 45 dots) [19]. We proposed that grouping might influence numerosity coding by changing the numerical units’ representation. However, we cannot exclude the possibility that the distance of inter-dots or the total area of dots might account for the decrease because clustering decreases the distance of inter-dots and increases the overlapping area of dots, which was proposed by the occupancy model [15,19]. We cannot exclusively identify object representation, which occurs at a higher level, as the only reason that grouping by clustering affects numerosity cognition because grouping is likely to occur at all levels of processing.

However, the potential explanations for distance or area can be completely ruled out in the current study. The distance, area, and other primitive visual characteristics similarly changed after clustering in the different awareness conditions and could not account for the distinct effects of grouping on numerosity processing. Therefore, the only plausible explanation is that grouping with awareness changed the object representation that defined the unit of numerosity coding and then affected numerosity processing. 

Previous research reported that object representation profoundly depends on neural mechanisms associated with awareness [20]. Without awareness, grouping cannot provide information used by the visual system to adjust its response and form a new object representation. Thus, the number of these new objects would not be represented, explaining why adaptation was not sensitive when dots in the current study were clustered without awareness. In short, grouping (by clustering) affects numerosity cognition by affecting object representation at a high-level of processing although grouping can occur in multiple levels and be achieved solely based on perceptual features.

### Duality in numerosity processing mechanism

 Monoptic and dichoptic masking are proposed to selectively affect the adaptation of distinct processing levels [20,38]. The adaptation of low-level properties, which is usually sensitive to early neural activities and is independent of awareness, is likely to be profoundly suppressed under the monoptic-mask condition [20]. By contrast, the adaptation of high-level properties, which is less dependent on early neural responses to adaptors and closely correlated with the mechanisms associated with awareness, may disappear under the dichoptic-mask condition, accompanying with the elimination of visual awareness [20,30]. However, numerosity adaptation existed under both conditions. A duality in numerosity processing is indicated, and it is well supported by the distinction in interocular transfer and “grouping effect” for numerosity adaptation with and without awareness. Thus, a parallel activating mechanism of low- and high-level processing in numerosity cognition [16] and the role of awareness should be considered.

Numerosity processing begins with processes of primitive non-numerical properties because numerosity information must be abstracted from visual properties. The processing of surrogate features must eventually lead to abstracted representations of numbers, and the latter should provide a meaningful presentation that is effective for the following processing. The visual system should first choose the numerical units to ensure this goal. Duality emerged because the discrete dots and the organized chunks can be candidates for the numerical units in our study. Representation of the former can be achieved by simulated retina activity [3,16], whereas coding of the latter should be additionally involved in high-level processing, such as perceptual organization, depth perception [30,45], or object representation [3,14]. Both a low- and a high-level approach can accomplish numerosity cognition, and the respective units would be chosen for different approaches. Our study showed that awareness is important in the engagement of distinct mechanisms in numerosity cognition because the absence of awareness would suppress high-level processing. 

Our study proposes that numerosity cognition without awareness is primitive, suggesting that numerosity cognition can be achieved by low-level processing. We do not believe that this feature challenges previously reported results [3,14,46–48]. Although low- and high-level stages may operate in parallel for numerosity cognition [16,49], we notably do not bother with choosing the numerical units in the presence of dualities. The cognition system automatically selects chunks (rather than discrete dots) to be the numerical units because clustering produces a more “global” level for each unit [3,46]. The evolutionary explanation of this representation in numerosity cognition is acceptable. Although we have a direct visual sense of the *sixishness* of cherries, similar to their *reddishness* [8], we do not need to know the number of speckles when we estimate the number of speckled hens. The units of a “local” level may be insufficient to generate reliable estimates in naturalistic situations that often employ objects with complex part structures during estimation [3]. Thus, objects in a more global level would be chosen to serve as units to approximate numbers, suggesting that high-level processing such as object representation is necessary in numerosity cognition with awareness. Our findings emphasize the crucial statuses of awareness and object representation in numerosity cognition and support the theory that processing of non-symbolic numerosity properties involves high-level cognitive stages.

## Conclusions

The numerosity property of adaptors can be adapted even when participants are unaware of the adaptors. Distinct mechanisms exist under different awareness conditions. The numerosity adaptation aftereffects were primarily binocular and affected by clustering of dots when participants were aware of the adaptors, whereas the adaptation aftereffects were primarily monocular and unaffected by grouping when participants were unaware of the adaptors. Our study shed light on the importance of awareness in numerosity cognition. With awareness, grouping (by clustering) affects numerosity cognition by changing object representations, and numerosity adaptation involves higher-level cognitive processing. 
